# Policy entrepreneurship and policy networks in healthcare systems – the case of Israel’s pediatric dentistry reform

**DOI:** 10.1186/s13584-017-0146-3

**Published:** 2017-04-21

**Authors:** Nissim Cohen, Tuvia Horev

**Affiliations:** 1 Department of Public Administration & Policy, School of Political Sciences, The University of Haifa, Haifa, 31905 Israel; 2 Department of Health Systems Management, Ben-Gurion University of the Negev, P.O. Box 653, Beer Sheva, 84105 Israel

**Keywords:** Policy entrepreneurs, Policy networks, Policy change, Pediatric dentistry reform

## Abstract

**Background:**

Can the entry of a policy entrepreneur challenge the equilibrium of a policy network and promote changes that might clash with the goals of powerful civil-servants and/or interest groups and, if so, why and how? Our goal is to examine two sides of the same coin: how does an in-depth analysis of Israel’s dental care reform enrich our understanding of policy networks and policy entrepreneurship? Second, how does the literature on policy networks and policy entrepreneurship help us understand this reform? Based on a theoretical framework that appears in the literature of policy entrepreneurship and policy networks, we analyze the motivations, goals and strategies of the main actors involved in the process of reforming pediatric dental care in Israel. We demonstrate how a policy entrepreneur navigated within a policy network and managed to promote a reform that, until his appearance, no one else in that network had succeeded in enacting.

**Methods:**

Our goals are advanced through a case study of a reform in pediatric dentistry implemented in Israel in 2010. It rests on textual analyses of the literature, reports, committee minutes, parliamentary proceedings, print and online media, and updates in relevant legislation and case law between 2009 and 2015. In addition, the case study draws on the insights of one of the authors (TH), who played a role in the reform process.

**Results:**

Historical circumstances and the Israeli public’s longstanding lack of interest in changing the existing model as well as interest groups that preferred the dominance of the private sector in the dental healthcare system kept that area out of the services supplied, universally, under the National Health Insurance Law. This situation changed significantly following the publication in 2007 of a policy analysis that contributed to shifts in the motivations and balance of power within the policy network, which in turn prepared the ground for a policy change. In this environment a determined policy entrepreneur, who identified a window of opportunity, took the lead and instituted an innovative and far-reaching reform.

**Conclusions:**

A policy entrepreneur can leverage external factors as well as the previous activities of a policy network that has already matured to create a policy change. Such entrepreneurial activity includes maneuvering around opponents and overcoming resistance from various stakeholders.

**Electronic supplementary material:**

The online version of this article (doi:10.1186/s13584-017-0146-3) contains supplementary material, which is available to authorized users.

## Background

The policy-network approach has been gaining ground since the 1970s as an important mainstream construct in the analysis of public policy [1–6]. According to this view, public policy is based on clusters of intra-governmental and non-governmental actors who typically maintain relatively stable ongoing relations and share interests and resources [7, 8]. These individuals are linked, both formally and informally, by rules that have become entrenched and institutionalized over time. Although they sometimes have divergent interests, they depend on each other and occasionally share common ground. The essence of their relationship is the surmounting of collective problems associated with what they perceive as acts of commission or omission, usually at the central government level, [9] and the promotion of specific policies that many or most of them consider desirable. The increased use of the term “policy networks” reflects the reciprocal relations and growing dependency between government officials (politicians and civil-servants from various agencies) and interest groups. Since power is decentralized and not exclusive to anyone, a network of contacts and interrelations takes shape that reflects the interests of each actor and the power relations among them in a given policy field.

As in many countries around the world, the civil-servants in the Israeli Ministry of Finance are considered powerful actors in the public policy arena. Despite their important role and the fact that without them it would be very difficult to maintain a responsible and balanced budget, their tremendous political power and controversial conduct have aroused a great deal of criticism [10–12].

Other powerful actors in the public policy arena are professional interest groups, which are composed of individuals who share a professional affiliation and act in concert to influence policy outcomes. The principal difference between such groups and “ordinary” policy groups is that it is usually their professional affiliation that generates the interest that its members share, making them a powerful influence in their area. In addition to their organizational ability, professional interest groups have more resources related to their area of expertise than anyone else in the network. These groups often manage to influence the design of policies that are consistent with their members’ interests by utilizing exchange relations and mobilizing other network members to support actions in the direction that they wish, often invoking the members’ goodwill and expertise. Thus, these groups frequently enjoy high status in policy groups [13].

Despite their power and expertise, members of a policy network—including the Ministry of Finance’s civil-servants and professional interest groups—sometimes fail to enact the public policy they desire even when facing policy reforms that are deleterious to their material or organizational interests. The explanation for this situation lies in occasional changes in the composition of the policy network and the balance of power between each individual or group and the other members of the network.

**Policy entrepreneurs** *Policy entrepreneurs are individuals who exploit opportunities to influence policy outcomes to promote their goals – without having the necessary resources required for achieving this goal alone [14–18]. They are not satisfied with merely promoting their objectives within institutions that others have established. Rather, they try to influence a given reality to create new horizons of opportunity for a policy change, using innovative ideas and strategies. These persistent individuals use innovative ideas and non-traditional strategies to promote desired policy outcomes. Whether they come from the private, public or third sectors, one of their defining characteristics is their willingness to invest their resources – time, energy, reputations and sometimes money – in the hopes of a future return [14]. This definition accords with Kingdon’s [15] and Mintrom and Vergari’s [19] suggestion that like their business counterparts, policy entrepreneurs are identifiable primarily by the *actions* they take, rather than by the positions they hold. However, given that actions are a function of motivation and ability, these elements are included within the definition of policy entrepreneurs. Hence, policy entrepreneurs, by definition, will always act to influence public policy processes. However, their ideas will not always necessarily be designed to bring about dynamic policy change. Based on their own goals, policy entrepreneurs will try to influence the amount of change, or even try to block attempts at change made by others. Thus, when it comes to politicians, we will determine whether or not they can be defined as policy entrepreneurs not only according to their dominance in the policy drama, but also based on their creativity, innovative strategies and untraditional activities. However, we will categorize them as policy entrepreneurs only if the policy they are seeking to enact goes beyond the traditional actions of elected officials. Note, too, that initiatives taken by a single member of the network will not be considered entrepreneurship unless his/her action changes the situation in accordance with the above-mentioned characteristics.* A note: As political science is not an exact science, scholars could reasonably differ somewhat in how they define and use the term “policy entrepreneur” [14].

### Policy entrepreneurs as destabilizing agents of change in policy networks

While policy entrepreneurs are not involved in most policy changes that occur worldwide, in many cases one cannot fully understand or explain policy outcomes without considering the role of policy entrepreneurs in actions that result in new policy outcomes. The 1990s yielded many theoretical insights into the motives and strategies that typify policy entrepreneurs [20–22]. They have been characterized as having a keen understanding of the society in which they operate and the ability to detect windows of opportunity for introducing solutions to social needs. They know how to define problems in a way that determines who will pay attention to them and know how to cope with them. Finally, they excel at team building and working with others effectively, and have enough knowledge and skill to engineer the change they seek. Furthermore, they often lead by personal example in translating ideas into concrete actions. By acting as pioneers in the field and mitigating decision-makers’ perceptions of risk, they make the change more palatable [23, 24].

Although policy networks may preserve a long period of stability or even stagnation, the traditional connections within a network can, and do change. Hence, over time, policy outcomes may change with them. The literature distinguishes between endogenous events, which occur within the network itself, and exogenous ones such as variables that may influence public policy. Smith [25] explains that exogenous variables change the way policy stakeholders perceive reality and, in turn, the way members of a policy network view the processes of policy assessment. Such a change may yield either an alteration in the composition of the network or a window of opportunity during which new network members may influence public policy. Similarly, Marsh and Rhodes [26] note that various changes in institutional, ideological, economic, or even technological factors may restructure the network, affecting policy outcomes and undoing realities that prevailed for years.

A policy entrepreneur who becomes involved in an environment typified by stagnation and permanent actors may affect rapid policy change by using entrepreneurial strategies to subvert the network’s old order and working assumptions. Given that a policy entrepreneur identifies sensibilities in society with a keener eye than others in the network, s/he will successfully detect windows of opportunity for change. The new entrepreneur in the network will redefine problems but not take on the rest of the network single-handedly due to the lack of the requisite knowledge, expertise, and resources. Therefore, newly arrived policy entrepreneurs rely on experts. They set up their own teams and coalitions of actors within and outside the existing network to engineer the change with the help of the latter’s knowledge, goodwill, and energy.

We argue that given the existence of a window of opportunity, a policy entrepreneurs who enter an existing network and have a unique status and motives may promote and produce rapid and meaningful policy change even in the face of resistance from significant actors. Nevertheless, despite their entrepreneurial abilities, when it comes to a significant change at the national level, policy entrepreneurs must have a critical mass of political power or authority to leverage existing windows of opportunity and implement their strategies after considering *when and how* to use their political power or authority.

## Methods

We test and substantiate the above mentioned contention through a case study of a reform in pediatric dentistry implemented in Israel in 2010. It rests on textual analyses of the literature, reports, committee minutes, parliamentary proceedings, print and online media, and updates in relevant legislation and case law between 2009 and 2015. In addition, the case study draws on the insights of one of the authors (TH), who played a role in the reform process.

### The Justification for choosing the case study

We chose this case study because of a long-standing anomaly in Israel’s oral-health system. While the country’s healthcare system at large evolved on the basis of a Bismarckian social-democratic model, the dental-health system operated on totally different principles that favored private dentists and commercial enterprises, nearly all paid for by the patients themselves. This fact is due to historical circumstances. An organized dental system in Israel was established in 1918 as an initiative of numerous dentists, members of a delegation from Hadassah, an American Zionist organization. Even though Hadassah tried to establish few dental clinics near hospitals, they were closed shortly afterwards, due to budget constraints. In 1923, Kupat Holim Clalit, the leading national health insurance provider that offered services to its members throughout the country, separated dental services from the rest of the health insurance it provided at that time. Therefore, most of the dental system evolved as a private system. A lack of power and political will among decision makers kept this situation in place, despite the establishment of a public healthcare system in Israel. Dentistry remained unregulated and lacked meaningful public funding [27].

## Results

### The case study^1^

In 2009, the government decided to triple the public budget for the delivery of preventive dental services to schoolchildren, administered by municipal authorities and under the responsibility of the Ministry of Health (MOH) [28]. In July 2010, a major watershed was crossed when the National Health Insurance Law (NHIL) was amended to add preventive and restorative (e.g., fillings) dental-health services for children to the package of insured services [29]. This law integrated dental care for children with the rest of the services delivered by the country’s four health plans (corresponding to HMOs) to their members based on public funding.

As we will demonstrate, unlike most of the changes within the Israeli health care policy, the pediatric-dental reform underscores the importance of *individual* agency as a promoter of policy outcomes. Hence, this case was *not* a traditional one of a politician promoting a desirable change in policy. Rather, this was an act of policy entrepreneurship, who entered an active policy network, which prompts us to move beyond mainstream notions of institutions in equilibrium.

### Emergence of the issue – the early stage

In 1988, a state investigative commission was established to examine the functioning and efficacy of the Israeli national healthcare system. The commission’s report, published in 1990, included, among others, a recommendation to include pediatric dental care in the list of services of a NHIL (National Health Insurance Law), once such a law was approved [30]. Nevertheless, when the NHIIL passed in 1994, [31] restorative dental care for children was not included in the list of services that should be provided by the health funds (listed in the Second Addendum of the NHIL). As noted in the Additional file 1, prior to the enactment of the NHIL, the Israel Dental Association (IDA) was one of the opponents to including dental services in the NHIL. At the time, some academics [32–36] and politicians [37] discussed the inefficiencies of the dental healthcare system as well as the importance of including these services in the NHIL, but it received little public attention. A major change in the public and legislators’ consciousness occurred pursuant to 2007 publications written by researchers of a social-policy think tank [38, 39]. As described in the Additional file 1, one of the argument that appeared in these publications was that the MOH was violating its responsibility under the NHIL to provide dental services for schoolchildren universally. The authors also highlighted the problems involved in leaving oral and dental health to the largely unregulated private market and emphasized the worst inefficiencies in the existing system. They proposed two independent paths for the government to solve some of the inefficiencies. Given a dormant policy-network that existed prior the publication and the extensive activities in civil-organizations, Knesset and the government that followed the publications and their impact, these publications might be considered as a turning point in the process of a policy change.

### Awakening the policy network

An initial attempt was made in 2008, within the MOH, to suggest universal coverage for pediatric dentistry as well as for the elderly population under the NHIL, but it failed. It did not receive the support of the Ministry of Finance (MOF) and was not discussed in the government [40]. It worth mentioning that a continued commitment to this issue was expressed later by the Director General (DG) of the MOH (in June 2009), who publicly expressed his views regarding the justification for including dental treatment in the NHIL (in addition to two other reforms he wanted to promote), mentioning the previous failed attempt to implement it.

Based on the information and provocative arguments presented in these publications [38, 39], in 2008 three civic organizations submitted two petitions to the High Court of Justice (HCJ) regarding dental health services for schoolchildren [41]. They claimed that the MOH provides these services in a discriminatory manner since the service is not accessible in many localities in Israel, and that in doing so, the ministry was violating the legal obligation imposed on the state since 1995 by the NHIL to provide dental services to all schoolchildren in Israel (as listed in the Third Addendum of the NHIL). Ostensibly, a positive decision of the court might have had serious implications such as class action suits, so the state had an interest in avoiding a court ruling. A few month later (November 27th, 2008), a coalition of 12 civic organizations was established to promote the provision of public dental services (preventive and restorative treatments) by the HMOs under the NHIL^2^. In addition, after many years with no bills in the Israeli parliament (the Knesset) regarding universal public coverage for pediatric dentistry, between 2008 and 2009, 15 private-member bills about dental care were proposed, some of them specifically proposing the inclusion of pediatric dentistry in the NHIL [42].

### Entrance of a policy entrepreneur

On April 7, 2009, Knesset member Rabbi Yaakov Litzman was named as a new Deputy Minister of Health (hereinafter: the DM or the entrepreneur). He joined the coalition as a delegate from Yahadut Hatorah, a sectorial party that represents part of Israel’s ultra-Orthodox minority. According to his party’s coalition agreement with the ruling Likud party, [43] the Prime Minister would hold the health portfolio throughout the government’s term, and a representative of the Yahadut Hatorah party would serve as sole deputy minister in the MOH^3^. The Prime Minister, the sides also stipulated, would empower the DM to act in his name in the Knesset and in the MOH in all health ministry-related affairs within his competence and remit. As opposed to other social services, in this agreement the Yahadut Hatorah party raised no demands regarding dental-health services. As explained in Additional file 1, and given his statement upon entering the MOH, [44] the DM seemed to have no previous intentions about championing the issue of pediatric dentistry when he took office.

### Agenda setting

One month prior the DM’s entrance to the MOH, a senior researcher who had promoted dental services in the NHIL since the mid-1990s and initiated the above-mentioned papers that led to a petition to the HCJ, [38, 39] left his position in a think tank and moved to the MOH as a Deputy Director General (hereinafter: DDG). At the beginning of June 2009, the DM and the Director General of the MOH received a position paper from the DDG stressing the need to include pediatric dental care in the NHIL. The paper described several arguments that might be interpreted as a window of opportunity for change. He pointed to the process for annual additions of new medical technologies and services to the basic basket of services supplied under the NHIL, as a possible platform. However, he suggested that the decision about whether to add pediatric dentistry to the NHIL benefits package should be decided by the DM and should not be not be prioritized by others (such as a designated committee for prioratizating).

Shortly afterwards, apparently based on the position paper as well as other activities described above, the DM announced his intention to carry out a plan to include dental health in the NHIL, using part of a budget originally designated for the inclusion of life-saving technologies (hereinafter: LST) [45] in the basic basket of the NHIL. He was subsequently backed by a government resolution [46].

### Interactions between the members of the network

Given activities made by the civil society coalition, the increased interest in, and legislators’ awareness of, the issue, all of the actors in the relevant policy network supported the idea of public funding for pediatric dentistry and its inclusion in the NHIL, including professional interest groups such as the IDA. However, several of them raised objections about two issues: the funding source of the reform (cutting a budget designated originally for LST) and the service-provision model of the reform (whether or not to limit the provision only to the existing HMOs that provide most of the services under the NHIL). With regard to the first issue, in December 2009 three petitions were submitted to the High Court of Justice (HCJ) (one by the Israel Medical Association, IMA, and others by two other civic organizations) against the government resolution cutting the budget for LST in order to fund the reform [47]. Civic organizations were divided on the issue. Some of them supported the petitions against the government, but others joined the suit as *amicus curiae* of the HCJ and defended the government’s resolution [48]. In May 2010, the court announced that the government resolution enabling cutting the budget for LST to fund the pediatric dental reform was nullified due to procedural errors in the legal approval of the decision. This procedural problem, that had to be corrected, made it unnecessary for the court to address the government’s intention to use a designated MOH budget for funding pediatric dental care on a substantive level [49]. As described in the Additional file 1, the procedural error was subsequently corrected, and with that the legal obstacle for approving the government decision was removed. The money was then transferred to fund the reform, as originally planned.

As for the second issue (the service-provision model of the reform), the MOF and the IDA shared a common interest. The latter favored the establishment of an independent Corporation (or authority) to Deliver Dental Services under the NHIL (hereinafter: CDDS) - as a substitute for the HMOs or in addition to them. The CDDS was supposed to augment as many independent dentists as possible [50]. The MOF saw the proposed CDDS as an opportunity to increase competition between public insurers (the HMOs), hoping that later on it could expand its responsibilities and turn it into a fifth HMO [51, 52]. The HMOs, civil organizations and most MOH senior-officials objected to this model. As described in the Additional file 1, this model gained wide support among members of the government, including the Prime Minister. The DM decided to support the CDDS model. Discussions in the Knesset on the proposed bill resulted in approval of the bill and the concept of a CDDS (in addition to the current HMOs), but limited the approval of the CDDS to ‘corporations for public benefit’ (non-profit organizations) [29]. Consequently, very few candidates have applied and so far, none have been approved.

The first phase of the reform was implemented on July 1, 2010 and included all children up to 8 years old [29, 53]. They were entitled to receive dental services through their HMO (no copayments on preventive care and minimal copayments on restorative services). In subsequent years, the entitlement was to be expanded, adding two cohorts of children each year. The last stage was scheduled for 2013, expanding the entitlement to children 12–14 years old [54].

As for the petitions to the HCJ mentioned earlier [41] based on the Third Addendum regarding universal provision of dental services to schoolchildren by the state through the local authorities, there were long delays by the court in handing down its ruling. A suspension of legal proceedings can sometimes encourage action at the executive authority level and obviate the need for a court decision, as indeed happened. Only in September 2010, about 3 months after the first phase of the pediatric dentistry reform had been implemented, did the HCJ conclude that - given that the state had budgeted 30 million NIS for preventive dental services for schoolchildren, was working to provide them through all local authorities, had included dental services for children as an integral part of the Second Addendum of the NHIL [provided by the HMOs] and was committed to expanding the program to all children up to 14 years old, “Under these circumstances, the circumstances that existed at the time of the filing of the petitions have changed and the appeals have been exhausted.... therefore, they are annulled…” [55].

In July 2011, the entitlement under the NHIL was expanded to children up to 10 years old and in July 2010 to children up to 12 years old, as originally planned.

### Importance of formal authority in public policy entrepreneurship

In March 2013, following general elections, the government was replaced. The new Minster of Health and officials of the MOF canceled previous inter-Ministerial agreements and did not implement the last stage of the reform (expansion of the entitlement to children up to 14 years of age) [56]. As described in the Additional file 1, apparently, faced with the need to decide where to invest the limited public funding allocated for the healthcare system, and since the new minister, apparently, did not include the promotion of oral health among her top priorities, she decided not to implement the last stage of the pediatric reform. In July 2014, the former DM, being a member of the Knesset with no governmental position, proposed a private bill (backed by 40 MKs) to include dental services for children up to 18 years old in the NHIL [57]. This initiative failed [58].

On May 14, 2015, following general elections, Israel changed governments again. Although the former DM returned to the Ministry of Health as a DM, 4 months later he was nominated to be a minister (following a ruling by the HCJ requiring “a full time minister” to be in the MOH). As opposed to the former agreement, this time expanding the entitlement to pediatric dental care was one of the demands raised by the Yahadut Hatorah party, which appeared in the coalition agreement they signed with the ruling Likud party. The new Minister (the former DM) implemented the last stage of the reform, and consequently since January 2016 all children up to 14 years old have been eligible for pediatric dental care under the NHIL, as was initially intended [59].

In addition to the pediatric dental services provided by the HMOs, preventive check-ups in oral health as well as oral health education lessons are provided to schoolchildren by most of the local authorities in Israel under the responsibility of the MOH [60].

## Discussion

We opened with the question of whether the entry of a policy entrepreneur can challenge the equilibrium of a policy network and promote changes that might harm the interests of powerful interest groups and civil-servants and, if so, how? Our study illustrates that policy entrepreneurs may promote a policy change *not only* because of events outside the policy network but also due to changes within it. In our case study, researchers who became brokers of change-first, by publishing provocative policy papers calling for a significant policy change that was then leveraged by civic organizations [61] who challenged the established order, appealed to the HCJ, and increased public and media awareness; and second, by becoming later a governmental player in the Ministry of Health. The HMOs entered the arena by offering free pediatric dental services in their supplementary insurance and became active stakeholders, who had the knowhow and the needed infrastructure to supply the service, once it would be included in NHIL. The same MOF, that had refused to promote such a reform in the past, now faced new incentives to reconsider its stance, due to a looming threat following petitions submitted to the HCJ. The maturation of the coalition activities conducted in the policy network prior to the entrepreneur’s appearance created a promising environment for a policy change.

In our case, we might conclude that the above-mentioned publications, [38, 39] together with actions by civic groups that followed them, led to increased public and media exposure to the situation in children’s oral health, helped to recruit the legislators and to submit petitions to the HCJ, that involved the judiciary in the issue. A positive decision from the court might have had serious implications such as class action suits. Therefore, the state had an interest in avoiding a court ruling, which affected the former’s incentives for a policy change. Indeed, previous studies have recognized both the legislature and judiciary as two dominant factors that are influential in the policy design of Israel’s healthcare system [62–64].

Although all of these factors paved the way for a change in policy, and the network was already ripe for change, such action did not occur, mainly due to the lack of authority and power to create the change. It was not until a policy entrepreneur appeared on the scene, a new member of the network who identified a window of opportunity and used his authority, that it was possible to alter the situation.

As mentioned, politicians who are positioned in the government can be considered policy entrepreneurs based on their actions, but only as long as these actions not only demonstrate dominance in the policy arena, but also go beyond the traditional framework of their activities in the government and promote innovative ideas and/or strategies. Two examples might demonstrate these characteristics in the case of the DM (for more details, see Additional file 1). The first was his controversial decision to use part of the LST budget as a funding source for the pediatric reform, overcoming objections from many powerful members of the policy network. This decision was revolutionary in Israeli terms. It was unusual for a health minister to take a portion of an earmarked-budget designated for new technologies, and then use it to fund the inclusion in NHIL of a service singled out by the DM, thereby bypassing the public committee appointed to prioritize hundreds of technologies and services. The second was his willingness to adopt a very controversial service-delivery model (based on HMOs and CDDS), again, overcoming in-house and outside objections, and against recommendation of a statutory advisory committee (the Health Council), to implement the reform. These examples that combine the ability to recognize a window of opportunity, the use of innovative and untraditional strategies, together with his determination and persistence might be considered characteristics of a policy entrepreneur.

We also illustrated the potentially important role of a support system such as a professional infrastructure that backs the entrepreneur’s activity and helps him/her identify the window of opportunity, when such exists. Such a support system can help prepare detailed plans and present alternatives when pressure from interest groups or external circumstances require a deviation from the original plan. Sympathetic advocacy coalitions and support from other stakeholders in the policy network are also essential components of the safety net [65].

Our case also demonstrates that when public support, professional and political safety nets, the right incentives for key actors, and a window of opportunity exist, the entrepreneur can more easily overcome or persuade opponents. In this case, the large public and governmental consensus led professional interest groups to support the core ideas of the suggested reform. Rather than struggling to ensure their interests by opposing the core values of the new policy, they focused their efforts on reducing the potential damage to their interests through what might be considered secondary-level changes in the policy design (e.g., the service-delivery model).

In addition, our case illustrates *how* policy entrepreneurs can use their source of power and authority to promote change. The literature notes the importance of timing, the ability to identify a window of opportunity, persuasion skills and other personal characteristics of the entrepreneur and the techniques s/he uses as necessary elements of the policy entrepreneur’s toolbox [19–22]. Indeed, the entrepreneur in our case study cooperated and negotiated with both the MOF officials and the HMOs, while allaying the fears of interest groups such as the IDA and IMA. Nevertheless, our case also underscores that the entrepreneur’s relative strength in the policy network and in the government, along with his/her formal authority or the ability to leverage the formal authority of others are the most significant resources that determine the ability to create and maintain change. In the current study, the entrepreneur had all of the virtues described in the literature [14, 15, 17]. Nevertheless, after he stepped down, the new Health Minister, joined by officials in the Finance Ministry, canceled the last implementation stage of the original pediatric dental reform, and no other actors in the network were able to rally against that decision. The entrepreneur (the former DM) tried to do so from his position as a legislator in the Knesset through a private bill, [57] but he failed, even though no change had occurred in the basic rationale for the reform and even though he was joined by many Knesset members and supported by an advocacy coalition that remained intact. However, two years later, upon his return to the Ministry of Health, he could complete the reform, even though some external incentives such as the HCJ petition and the idea of adding a competitor to the HMOs (CDDS) that contributed to driving the reform initially no longer existed. This situation may have arisen not only because of the entrepreneur’s cumulative experience in the policy domain, but also because he returned to the government with greater power and authority than he had in the previous term, mainly because of the stipulation that appeared in the coalition agreement signed by his party, Yahadut Hatorah, and the ruling Likud party [66]. This time, shortly after his entrance, he was appointed as a Minister of Health (in contrast to his previous position as Deputy Minister)^4^.

In addition to the previously mentioned aspects of policy entrepreneurship behavior revealed in this case, another factor of importance is the consistent pressure from actors, who changed positions-inside and outside the government, but stayed committed to create the desired policy change. That characteristic behavior was also evident in the publications that analyzed other policy changes that ended in the enactment of Israel’s NHIL [67].

## Conclusions

In conclusion, the article described how a 2007 policy analysis served as an important catalyst to activities conducted by players inside and outside a policy network. In addition, on one hand, the paper strengthens and illustrates arguments in the literature about the factors within a policy network that might pave the way for a change. It demonstrates how the earlier activities of members of a network, including actions by the MOH, combined with a determined policy entrepreneur, can minimize the resistance of powerful civil-servants and professional interest groups in the network and channel their opposition to issues that are relatively easier to address. On the other hand, it highlights the impact of external events and authorities such as the judiciary in changing the interests and incentives of members of the network, and their crucial effect on the ability of a policy entrepreneur to promote a substantial policy change. However, it also emphasizes the enormous importance of the policy entrepreneur’s power and formal authority and the support systems within which s/he functions, as well as the political backing s/he receives that enables him/her to adopt a realistic agenda and implement a reform successfully.

## Additional file

Additional file 1: Collaboration and Struggles in Israel’s Dental-Health Policy Network: The Case Study [68–120]. (DOCX 52 kb)

## Abbreviations

CDDS: Corporation for delivering dental services
DDG: Deputy Director General
DM: Deputy Minister (of health)
HCJ: the High court of justice
HMO: Health maintenance organization
IDA: the Israel dental association
IMA: the Israel medical association
LST: Life-saving technologies
MOF: Ministry of finance
MOH: Ministry of health
NHIL: National health insurance law
NIS: Israeli New Shekel
NPO: Nonprofit organization

## Footnotes

1 See Additional file 1 for a detailed version of the case study.

2 The invitation letter for civil organizations to join the first meeting of founding the new coalition included a citation from the Taub Center publication mentioned earlier.

3 Throughout the years, due to ideological reasons, Yahadut Hatorah party refused to serve in the government and agreed only to serve as deputy ministers.

4 This happened following a decision of the Supreme Court that dismissed the possibility of appointing a Deputy Minister of Health, without a full-time minister. However, some argue that given the status and authority the DM had during his first term of office, from a practical standpoint, the former DM had essentially the same influence as a minister as he had as a deputy minister.

## References

[1] Van Waarden F (1992). Dimensions and types of policy networks. Eur J Polit Res.

[2] Sabatier PA, Dunn WN, Rita Mae K (1992). Political science and public policy: An assessment. Advances in Policy Studies since 1950.

[3] Bressers HTA, O’Toole LJ (1998). The selection of policy instruments: A network-based perspective. Journal of Public Policy.

[4] Thatcher M (1998). The development of policy network analyses: From modest origins to overarching frameworks. Journal of Theoretical Politics.

[5] Rhodes RAW (1997). Understanding Governance: Policy Networks, Governance, Reflexivity and Accountability.

[6] Bevir M, Rhodes RAW (2003). Interpreting British Governance.

[7] Laumann EO, Knoke D (1987). The Organizational State: Social Choice in National Policy.

[8] Peterson J (1995). Policy networks and European Union policy-making: A reply to Kassim. West European Politics.

[9] Borzel T (1998). Organizing Babylon—On the different conceptions of policy networks. Public Adm.

[10] Ben-Bassat A, Dahan M (2006). The Balance of Power in the Budgeting Process.

[11] Cohen N (2013). The power of expertise? Politician-Bureaucrat interactions, national budget transparency and the Israeli health care policy. Policy Studies.

[12] Cohen N (2015). Bargaining and informal interactions in the national budget: A game theory analysis of the Israeli case. Int Rev Adm Sci.

[13] Mitchell W, Munger M (1991). Economic models of interest groups: An introductory survey. Am J Polit Sci.

[14] Cohen N. Policy entrepreneurs and agenda setting. In Zahariadis, Nikolaos (ed.), *Handbook of Public Policy Agenda-Setting*. (2016); Edward Elgar, pp. 180–99.

[15] Cohen N (2012). Policy entrepreneurs and the design of public policy: Conceptual framework and the case of the National Health Insurance Law in Israel. Journal of Social Research & Policy.

[16] Mintrom M, Norman P (2009). Policy entrepreneurship and policy change. Policy Studies Journal.

[17] Navot D, Cohen N (2015). How policy entrepreneurs reduce corruption in Israel. Governance.

[18] Kingdon JW (1995). Agendas, Alternatives, and Public Policies.

[19] Mintrom M, Vergari S (1998). Policy networks and innovation diffusion: The case of state education reforms. J Polit.

[20] Cohen N, Naor M (2013). Reducing dependence on oil? How policy entrepreneurs utilize the national security agenda to recruit government support: The case of electric transportation in Israel. Energy Policy.

[21] Mintrom M (2013). Policy entrepreneurs and controversial science: Governing human embryonic stem cell research. Journal of European Public Policy.

[22] Arieli T, Cohen N (2013). Policy entrepreneurs and post-conflict cross-border cooperation: A conceptual framework and the Israeli-Jordanian case. Policy Sci.

[23] Schneider M, Teske P, Mintrom M (1995). Public Entrepreneurs: Agents for Change in American Government.

[24] Mintrom M (2000). Policy Entrepreneurs and School Choice.

[25] Smith MJ (1993). Pressure, Power and Policy. Harvester Wheatsheaf, Hemel Hempstead, Herts.

[26] Marsh D, Rhodes RAW, Rhodes RAW, Marsh D (1992). Policy networks in British government. Policy Networks in British Government.

[27] Shvarts S, Doron H (2009). Dental health for all: Activity of Clalit Health Services toward or the establishment of public dental medicine in Israel. Whither the Healthcare System?.

[28] Government Resolution no. 140. Section 3. May 12, 2009. Jerusalem: Israeli Government.

[29] National Health Insurance Law (2010). 50th Amendment.

[30] Israel Yearbook (1990). Report of the Netanyahu Commission, Chapter 6.

[31] National Health Insurance Law (1994). Law. 5754. Sefer Huqim, no.1469.

[32] Horev T (1996). The relation between dental morbidity and needs to dental health policy. Social Security (Isr).

[33] Berg A, Rosen B, Sgan Cohen B, Horev T (1996). Household expenditure on dental health.

[34] Horev T (1999). An optimal policy on dental health: Analysis of selected systems and proposal of an Israeli model.

[35] Horev T, Chernichovsky D (1999). An optimal dental health policy. Resource Allocation for Social Services.

[36] Berg A, Zusman SP, Horev T (2001). Social and economic aspects of dental medicine in the National Health Insurance Law.

[37] The Knesset (2000). Report of parliamentary investigative commission for examination of implementation of the National Health Insurance Law, Section 15. Chair: Member of Knesset David Tal.

[38] Horev T, Mann J (2007). Oral and dental medicine: The state’s responsibility to its residents.

[39] Horev T (2007). Delivery of healthcare services under the National Health Insurance Law—A requirement or a recommendation?.

[40] Y-net News. Meital Yasur-Beit Or. ‘Ministry of Health: Children and the elderly will receive free dental care’. In February 11, 2008. http://www.ynet.co.il/articles/0,7340,L-3505227,00.html (in Hebrew).

[41] HCJ 2311/08, Association for Civil Rights in Israel and Physicians for Human Rights *vs.* Ministry of Health, Ministry of Education, and Center for Local Government; HCJ 2422/08 Keren Dolev Foundation for Medical Justice *vs*. Minister of Health, Minister of Finance, Minister of Education, and Attorney General (in Hebrew); 2008.

[42] Knesset no. 17: Private Bills no. 3797/17 and 3926/17. Knesset no. 18: Private Bills no. 77/18; 264/18; 423/18; 740/18; 1131/18; 1163/18; 1231/18; 1291/18; 1398/18; 1469/18; 1480/18; 1546/18;1568/18. Based on information on the Knesset website. http://main.knesset.gov.il/Pages/default.aspx

[43] This is a coalition agreement between political parties. Coalition agreement between Yahadut Hatorah and Likud, signed and presented to the Knesset Secretariat by chair of Likud faction on April 1, 2009. (The Knesset endorsed the Government on March 31, 2009) (in Hebrew).

[44] Even D. Litzman: I came to rock the healthcare system. *Ha’aretz*, April 14, 2009. http://www.haaretz.co.il/news/politics/1.1255631

[45] Niv Shai. Globes. September 9, 2009. http://www.globes.co.il/news/article.aspx?did=1000496888 (in Hebrew).

[46] Government resolutions: no. 1064, from December 12, 2009; no. 1096 from December 20, 2009; and no. 1634 from May 2, 2010.

[47] HCJ. 10017/09 the Dolev Foundation for Medical Justice; HCJ 10094/09 The Movement for Quality Government in Israel (MQG); HCJ 10469/09 Israel Medical Association – all of the above - *v.* Government of Israel and the relevant ministers. (in Hebrew); 2009.

[48] HCJ. Appeal submitted to HCJ by: The Association for Human Rights in Israel, Physicians for Human Rights, Israel, and Adva Center – asking to join the court as ‘amicus curiae’, in petitions no. 10017/09; 10094/09; 10469/09. February 1, 2010.

[49] HCJ. High Court of Justice final resolution on petitions no. 10017/09, 10094/09 and 10469/09. Given on May 20, 2010.

[50] Israel Dental Association (IDA) (2009). National Health Insurance for Dental Medicine. A position paper.

[51] Amsterdamski S. A fifth sick fund is on the map, again. *Calcalist*. August 6, 2008. (in Hebrew). http://www.calcalist.co.il/articles/0,7340,L-3100184,00.html

[52] Minutes of the Knesset Labor, Welfare, and Health Committee (2010). Subject: National Health Insurance Bill no. 49. Meetings on July 5, 2010, and July 19, 2010 (protocol no. 333).

[53] NHI Order (2010). ‘A change in the second addendum of NHIL (no. 3) -2010’; see also NHI Order. ‘Adding an area in the first addendum of NHIL -2010’. both orders signed on June 14, 2010, by the PM and Minister of Health. Israel’s booklet of regulations no. 6901.

[54] Jerusalem: Israeli Government. Government resolution no. 2081. July 15, 2010.

[55] High Court of Justice (2010). Final resolution on petitions no. 2311/08 and 2422/08. Given on September 15, 2010.

[56] Example of responses to that decision: Linder-Gantz R. *The Marker* newspaper. August 5, 2013. http://www.themarker.com/consumer/health/1.2014737. Number of children, as appeared in the appendix, was calculated based on Israel Central Bureau of Statistics data. 2014. Jerusalem.

[57] Jerusalem: Sefer Huqim. National Health Insurance Law (a proposed bill regarding dental care for children up to 18 years), 2014, P-2656/19.

[58] The Israeli Government. Government resolution no. 2302 from December 11, 2014 and resolution no. HK/1241 of the government’s ministerial committee for enactment, from November 23, 2014.

[59] Jerusalem: Israeli Government. Government resolution no. 863, December 20, 2015.

[60] According to the Ministry of Health data, 220 local authorities (out of 255) offer this service to about 1.7 million children (age 5–14); 2015.

[61] As examples of their activities, see position paper from September 21, 2009 regarding dental health services in the NHIL, in: http://info.org.il/teeth4all/?page_id=6-and a position paper on oral health for the elderly population (2012): http://info.org.il/teeth4all/wp-content/uploads/2012/01/TeethReport_2011.pdf. In 2011–2012 they also promoted a private bill (p/18/3534) submitted on August 3, 2011 by 48 MKs regarding dental health services for the elderly population.

[62] Horev T (2004). Reflection of a healthcare policy in the mirror of legislation.

[63] Horev T, Bin-Nun G, Ofer G (2006). Court decisions and their impact on policy issues at the healthcare system: Aspects of the conflict between individual needs and public interest. Tenth anniversary of the National Health Insurance Law.

[64] Horev T (2005). Involvement of the judicial system in shaping healthcare policy. Med Law.

[65] Sabatier PA, Jenkins-Smith HC (1993). Policy Change and Learning: An Advocacy Coalition Approach.

[66] This is a coalition agreement betwen political parties. Coalition agreement for the formation of the 34th Government of the State of Israel, between Likud and Yahadut Hatorah, signed and presented on May 3, 2015 (Sections 13, 79) (in Hebrew).

[67] Horev T, Babad MY, Shvarts S (2003). Evolution of a healthcare reform: The Israeli experience. Int J Healthc Technol Manag.

[68] The Central Bureau of Statistics (CBS). Israel data (2009) were calculated based on data extracted from ICBS publication dated Sept. 10, 2015. Other countries’ data (2010) are based on data extracted from Eurostat database.

[69] Data on 2009 (2015). Extracted from OECD Health statistics.

[70] Data on 2002 (due to lack of data on 2009) (2015). Extracted from OECD Health statistics.

[71] World Health Organization. WHO Global Oral Health Database. http://www.who.int/oral_health/databases/global/en/

[72] Data on 2010 (2015). Extracted from OECD Health statistics.

[73] Israel CBS (2008). Calculations based on Households’ Expenditure Survey.

[74] Ministry of Health data (2007). Calculations were based on the National Social Survey. Israel CBS.

[75] Arian A, Shamir M (2002). Elections in Israel—2001.

[76] Cohen N, Mizrahi S, Yuval F (2011). Public attitudes toward the welfare state and public policy: The Israeli experience. Israel Affairs.

[77] Jerusalem: Sefer Huqim. National Health Insurance Bill (government), 1992. No. 2113. February 17, 1992. Section 2. p.211 (government #24).

[78] Jerusalem: Sefer Huqim. National Health Insurance Bill (government), 1993. No. 2189. June 30, 1993. Section 6. p. 207 (government #25).

[79] Jerusalem: Sefer Huqim. As examples of these discussions, see: Minutes of the 21st meeting of the Knesset’s joint commission for the enactment of the NHIL. November 30, 1993; see also MK Tamar Gojanski in Knesset discussions about 2nd and 3rd votes from Dec. 12 1994. Her remarks on dental health services did not raise any comments or discussions on the subject. Source: National Health Insurance Bill, 1994. Meeting no. 283 of the 13th Knesset. Dec. 12, 1994. Time-00:16. Jerusalem.

[80] Horev T (1999). Optimal policy on dental health in Israel. Lecture delivered at the first annual colloquium on healthcare policy.

[81] Nirel N, Horev T, Zusman SP, Rosen B (2003). Inequality in dental health in Israel. Background paper in final report.

[82] Horev T, Sgan Cohen H, Berg A, Rosen B (1996). Household expenditure on dental care.

[83] Horev T (2002). Governmental involvement in six industrialized countries’ oral healthcare systems.

[84] Horev T, Babad YM (2005). Healthcare reform implementation: Stakeholders and their role—the Israeli experience. Health Policy.

[85] Birkland TA (2011). An Introduction to the Policy Process: Theories, Concepts, and Models of Public Policy Making.

[86] Sperling D, Cohen N (2012). The influence of the Israeli state economy arrangement law and supreme court decisions on health policy and the right to health in Israel – Neo-institutional analysis. Hukim-The Israeli Journal on Legislation.

[87] Israel State Comptroller (2005). Report of the State Comptroller, 2004.

[88] Wilding P (1984). Professional Power and Social Welfare.

[89] Horev T, Kaidar N (2010). Light and Shadow in the Development and Implementation of the National Health Insurance Law: Reflection of the Reform Fifteen Years after Enactment.

[90] Machnes Y, Bin-Nun G, Ofer G (2006). Extent of efficiency and inefficiency in dental health. Tenth anniversary of the National Health Insurance Law.

[91] Reshumot (1995). *Official Gazette* (IP) 4288.

[92] Epstein L, Horev T (2007). Inequalities in the Israeli health care system: Presenting a national problem and a proposed policy to cope with it.

[93] Horev T (2008). Narrowing health inequalities: International experience and its implications for Israel.

[94] Israel Medical Association (IMA) (2008). Healthcare inequality in Israel.

[95] Y-net News. Meital Yasur-Beit Or. ‘Ministry of Health’s Director General: to include dental services in the NHIL’s basket of services’. June 4th, 2009. http://m.pplus.ynet.co.il/Article.aspx?id=3726022 (in Hebrew).

[96] Kop Y (2008). Strategies for narrowing socioeconomic gaps. Report of a task force appointed by the President of Israel in conjunction with the Minister of Welfare and Social Services.

[97] Ministry of Health. *For a healthy future. Oral and dental health in Israel, targets for 2020—final report.* 2009. See also project description on Ministry of Health Web site: http://www.health.gov.il/Subjects/HealthyIsrael/Pages/default.aspx, see also abstract on coalition Web site, http://info.org.il/teeth4all/?p=243.

[98] Navon G, Chernichovsky D (2008). Dental medicine: The burden of expenditure on households.

[99] Machnes Y, Carmeli A (2009). Provision of oral care by local government authorities in Israel: Policy issues and empirical evidence. Health Policy.

[100] The Coalition for Public Health Dentistry’s Internet site: http://info.org.il/teeth4all

[101] Jerusalem: Sefer Huqim. Examples of private bills submitted to the Knesset, prior the entrance of the Deputy Minister to MOH: Bill no. 3797, submitted on June 23, 2008 (18th Knesset), by Muhammad Barakeh and two other MKs; Bill no. 423, submitted in April 1, 2009 (19th Knesset), by Chaim Oron and two other MKs; Bill no. 264, submitted in April 1, 2009 (19th Knesset), by Ofir Pines.

[102] Meir Gold. Molar teeth. *HaModia*. Jerusalem: March 6, 2009 (in Hebrew).

[103] Bank of Israel Spokesperson’s Bureau. Press release: Adding pediatric dental care to the public basket. Preview of Bank of Israel annual report. April 13, 2010. Jerusalem: Bank of Israel, http://bankisrael.org.il/he/NewsAndPublications/PressReleases/Pages/100413t.aspx

[104] Cashman, GF. Taub director calls for new approach to socioeconomic gaps. Jerusalem: *The Jerusalem Post,* Dec. 22, 2009.

[105] Knesset. Minutes of the 21st meeting of the Knesset’s joint commission for the enactment of the NHIL. November 30, 1993.

[106] Linder-Ganz R. The government buried the dentistry reform. *Ha’aretz,* May 3, 2010 http://www.haaretz.co.il/misc/1.1200323

[107] The Knesset (2010). Minutes of Labor, Social Affairs, and Health Committee Meeting 294.

[108] MOH (2010). Minutes of the Health Council meeting.

[109] Knesset. *Divrei yemei ha-Knesset* (Knesset record). May 24–25, 2010 (in Hebrew). In addition, see the following citation from the NRG Internet newspaper, June 26, 2010. “Prime Minister Benjamin Netanyahu ordered Litzman that dental care would be provided for children from the start by HMOs with the assistance of private clinics and independents dentists. "It is important that the public receives the broadest and best service," Netanyahu said. "Therefore, enabling healthy and free competition in the provision of dental care for Israel’s children is important.”

[110] Government Bill no. 509 (2010). National Health Insurance Law (amendment no. 49).

[111] Sefer Huqim. Israel Companies Law 5759–1999; 1999.

[112] Government Resolution 1064, December 14, 2009, concerning the inclusion of dental care in National Health Insurance. http://www.pmo.gov.il/Secretary/GovDecisions/2009/Pages/des1064.aspx (in Hebrew).

[113] Knesset. This notion was also expressed by MKs who opposed the MOH’s decision in a meeting of the Knesset’s health lobby on November 10, 2009. It also appeared on the invitation to the meeting; 2009.

[114] National Institute for Health Policy and Health Services Research (2010). ‘Policy Forum’ meeting. Topic: “Dental health for all—when? At whose expense? How?”.

[115] NHI Order (2010). ‘Adding an area to the first Addendum of NHIL (A temporary provision) – 2010’. Signed on June 14, 2010, by the PM and Minister of Health. Israel’s booklet of regulations no. 6901.

[116] Israeli Government. Government resolution no. 1634 from May 2, 2010.

[117] Israeli Government. The announcement was based on Section 3 of government resolution no. 140, from May 12, 2009.

[118] Announcement of the government secretary from July 22, 2010 http://www.pmo.gov.il/mediacenter/spokesman/pages/spokegufim220710.aspx

[119] ‘Achievements of Israel’s government headed by Benjamin Netanyahu, after the last 4 years’ The Likud internet site: https://www.likud.org.il (in Hebrew)

[120] Ynet website. April 11, 2013. “Minister of health signed today the new regulations that forbid fluoridation of drinking water in Israel http://www.ynet.co.il/articles/0,7340,L-4366896,00.html

