# Supplementary material for: Policy entrepreneurship and policy networks in healthcare systems – the case of Israel’s pediatric dentistry reform
Source: Isr J Health Policy Res. 2017 Apr 21;6:24. doi: 10.1186/s13584-017-0146-3 (PMC5399377; doi:10.1186/s13584-017-0146-3)
Supplement: Additional file 1: — Collaboration and Struggles in Israel’s Dental-Health Policy Network: The Case Study [68–120]. (DOCX 52 kb) [file 13584_2017_146_MOESM1_ESM.docx]

**Additional file 1**

**Collaboration and Struggles in Israel’s Dental-Health Policy Network: The Case Study**

Historically, the dental healthcare system in Israel has been hardly regulated and was funded mostly by out of pocket payments. It was inefficient and not accessible to a large portion of the population. In the period preceding the reform (2009), the national expenditure on dentistry was approximately 8.4% of the total expenditure on health, higher than in countries such as Sweden (7.5%), Switzerland (6.3%) and the Netherlands (3.6%). [68] The density of practicing dentists per population in Israel at that time was 0.81 per 1,000, higher than the average of 21 OECD countries for which data existed (0.64). [69] Despite relatively high inputs and an adequate supply of dentists, dental morbidity, as reflected in the incidence of dental caries in Israel, was relatively high. For example, the DMFT index, which reflects the cumulative dental caries experience among children aged 12, in Israel was 1.7 - higher than in countries such as Denmark and Luxembourg (0.9), the Netherlands (0.8), Austria and Australia (1.0) and Sweden (1.1). [70]

Unlike European countries such as Germany, Sweden, Britain, Norway and Austria in which all citizens enjoy at least basic governmental funding for dental services, in Israel, dental treatments for children and the elderly [71] were not included in the basic basket of healthcare services. Government funding covered less than 1.5% of the national expenditure on out-patient dental care compared to the OECD average, which was 31.5% (26 countries), and far less than countries such as Sweden (40%), Germany (57%), Austria (49%), France (36%), and even the US (10%). In that sense, only one country (Spain) was in a worse situation than Israel. [72] Household spending on dental care in Israel accounted for 28.6% (2008) of the total household consumption expenditures on health. [73] High out of pocket payments created an economic barrier to dental care. Indeed, a national survey revealed that approximately 66% of respondents who earned less than the minimum wage in Israel, and 58% of those who earned between the minimum wage and the national average wage at the time, reported that during the previous year they gave up needed dental treatment due to its cost. [74] Insurance companies in Israel offer voluntary private dental insurance policies (mostly, under group insurance policies), but only about 8%-10% of the population has purchased such coverage, and it covers 9% of outpatient dental costs [72].

***Israel’s dormant dental-health policy network prior to the reform***

Historically and generally speaking, the Israeli public tends to take an indifferent and passive view of social policy issues. [15] Studies on Israeli society show that most Israeli citizens are more concerned about national defense and foreign policy than socio-economic issues. [75] Therefore, social issues that are of major concern in other countries were often marginalized in Israel. While most Israelis have come to believe that more government resources should be invested in healthcare than in defense, [76] even during a series of mass social protests that took place in 2011, health was not a major focus and dental health not at all. This phenomenon reinforces the assessment that the Israeli public, including its elected representatives, has not acknowledged the vital necessity of dentistry as a public service and tends to accept the situation as is. [27]

In 1988, a state investigative commission was established to examine the functioning and efficacy of the national healthcare system. The commission’s report, presented in 1990, included recommendations for reforms in various fields, central among which was the passage of a national health insurance law. Attached to the report was a minority opinion regarding several issues but not dentistry. On that topic, all of the committee members agreed that pediatric preventive and restorative dental care should be among the NHI services, once the law was approved. [30] Nevertheless, in spite the fact that in a National Health Insurance bill submitted in 1992, [77] dental treatment for children and the elderly population appeared as part of the services that were supposed to be covered under the suggested NHIL, that area did not appear in other bills that were proposed, nor in a later government version of the bill (1993) [78]. At that time, the IDA opposed even the suggestion of including preventive dental services for schoolchildren in the NHIL. [79] In the end, when the bill was approved in 1994, restorative dental treatments for children (and the elderly) were not included in the law. [31] However, preventive dentistry for schoolchildren that was provided by the state prior to the reform and appeared in all of the preliminary NHI bills did appear in the final version and was included in the law (and specified in the Third Addendum of the NHIL).

During the legislative procedures, very few discussions about pediatric dentistry in the NHIL took place. [79] In the years preceding and following the commission’s work, and until the NHI became law (1995), academics and politicians rarely addressed the topic of a national policy on dental health. Shortly after its passage, some academics and politicians began discussing the issue. [32-37] The topic was also included in scientific colloquia in Israel [80-82] and abroad, [83] all of which raised a modest degree of awareness, chiefly among those already interested in the matter, but did not precipitate a real change in the public’s awareness and the decision-makers’ motivations. As a result, no significant network interactions to promote a policy change occurred.

*Potential members of the pediatric-dentistry policy network*

Several major actors within and outside the government might interact within a policy network in a manner relevant to oral and dental health in Israel.

*Ministry of Health (MOH):* Ever since it was established, those involved in the Israeli healthcare policy arena have regarded the MOH as a ministry with relatively modest political influence. [11] Generally speaking, its senior dental professionals favored the inclusion of oral health in NHI services and in the past raised this idea, but they did not prioritize this issue.

*Ministry of Finance (MOF):* Finance ministry officials in all countries are considered bureaucratically important. [12] In the specific context of the Israeli healthcare system and NHIL, they are thought to be highly if not definitively dominant. [10, 11, 84] As explained elsewhere, [12] the officials in this office concentrate their efforts on restraining the annual government budget and usually take action in situations they perceive as inefficient that justify government intervention.

*The political system and the politicians:* For many years, advocating for changes in dental care was not a priority for most politicians. Even though a February 2000 report of a parliamentary commission that investigated the implementation and funding of the NHIL included a laconic recommendation to include dental health in the NHI services, [37] it did not change the situation. In 2003-2004 no bills regarding oral health were submitted in the Knesset, whilst during that period 64 bills on other health topics were proposed. Since 2005 several legislative proposals about dental issues have been made, mostly in regard to dental treatment for populations with special needs, but the Israeli politicians who promoted dental health were generally not dominant figures in the political system. No moves toward universal public coverage for pediatric dentistry were evident in the Knesset until 2008.

*The Supreme Court and the State Comptroller*: The Supreme Court (or The High Court of Justice (HCJ) is very influential with regard to policy adoption, and the professional literature both abroad [85] and in Israel [86] considers it a significant factor in the public policy arena. Recent studies in Israel note the HCJ’s favoring of rulings that promote neo-liberal policies in various domains including healthcare [86] and influence the administration and policy-decision process in the healthcare system. [63] Until 2008, the court did not address issues related to national dental health policy.

As for, the Israel State Comptroller, in 2005 an annual report included a chapter on dental-health services, noting that the MOH had not presented any alternatives for providing such services. [87] Once again, the report led to no changes in policy.

*The Israel Dental Association (IDA):* The IDA is a professional interest group. As a voluntary organization, its members and elected officials usually come from the private sector. As a rule, a profession influences the making of social policy by pressuring politicians and decision-makers, placing senior experts in their field in key public positions and leveraging their professional knowledge. [88] Until 2009, dentists held no senior posts that might influence government policy. Historically, the IDA as a professional organization has not played a major role in public healthcare policymaking. Nevertheless, that does not mean that the IDA's leaders were not involved and could not be influential in Israel's politics.

*Civic organizations:* Two important civic groups are of concern here: advocacy organizations and non-profit service providers. Organizations of the latter kind have been delivering dental care in Israel continually, largely for the needy and on a philanthropic basis, and sometimes subsidized by the state. Usually, they were not involved in public policymaking. In contrast, the advocacy organizations were very involved in public policy but tended to focus on social and health issues other than dental care. However, as described below, this focus shifted in the two years preceding the reform.

*HMOs and private health insurers*: Most healthcare services in Israel are delivered by four public, not-for-profit HMOs that operate on the basis of the NHIL. These institutions engage in controlled competition under MOH regulation. Since 1998, following one of the amendments in the NHIL, the HMOs were allowed to add services (such as dental healthcare) to their basic basket of services listed in the NHIL, voluntarily (upon approval), but none did so. However, they provided discounted dental treatments under their supplemental health insurance schemes (voluntary private health insurance).

In 2006, one of the HMOs (Meuhedet) intended to take the first steps towards the promotion of a dental insurance program under which it would be the policyholder of a group dental insurance policy offered by one of the insurance companies in Israel. However, due to personnel changes, the program was not activated. Soon after, another HMO (Maccabi Health Services) came out with a dental-health services plan for children up to age six, with no co-pay, as part of its second tier of supplemental health insurance schemes. Additional HMOs followed its lead. This venture proved to be a marketing success that convinced many young households to purchase the second tier of the HMO’s supplemental insurance. [89]

*Research institutes and academia:* In addition to researchers in academic institutions, think tanks, largely staffed by senior academic researchers as well as people who formerly held senior executive positions in the government, are regularly involved in the healthcare system simply because they focus on applied research and can study and present matters that they consider of national importance. However, until 2007, these think tanks contributed little to the discussion on national policy regarding oral health, and various articles that were published on this issue gained little public attention. [32-36, 90]

***Emergence of the issue and the rise of a policy network***

A major change in the public consciousness about the public coverage of dental care occurred pursuant to a 2007 study conducted by researchers in a social policy think tank. [38] It highlighted the problems involved in leaving oral and dental health to the largely unregulated private market and emphasized the most severe inefficiencies in the existing system. The publication evoked discussions by also proposing two independent paths for the government to solve some of the inefficiencies. The first was the introduction of dental services for children and the elderly to be delivered by the HMOs under the Second Addendum to the NHIL. The second path involved dental-health services for schoolchildren, which according to the Third Addendum to the NHIL are the responsibility of the MOH. The researchers called on the MOH to correct how it discharged its duties under the NHIL. One of the provocative arguments in this publication [38] and described in more detail in another publication, published shortly after the first one, [39] was that the MOH was violating its responsibility under the NHIL to provide dental services for schoolchildren universally (mostly, but not exclusively, preventive services), as stipulated in the Third Addendum of the NHIL. The basket of services and additional relevant details regarding how these services were supplied prior to the enactment of NHIL were also included in an order from the Minister of Health, published in 1995 (e.g., the dental services included two levels of service: the basic one - preventive, and the second one - restorative treatments). [91] However, prior to the enactment of the NHIL, it was the municipal authority that decided whether to provide these services and at what level. At that time, only a few municipal authorities provided these services under the supervision of the MOH. This situation continued after 1995, even though the new NHIL required universal coverage. Among the questions raised in that paper was: “If during the last decade, the state did not provide the service and apparently systematically refrained from doing so, then beyond the moral questions and economic consequences of this decision on the national level, there is also the question of compensation for the damage caused during all these years to the teeth of children in those local authorities that did not provide dental service.” [38]

In the wake of media coverage of these publications, additional studies substantiated the need for changes in dental health policy and the inclusion of this area in the basic NHI package under the HMOs’ responsibility, as one of the ways to mitigate inequality in the healthcare system [92-93]. One of the studies that made this point was a policy paper published by the IMA (Israel Medical Association). [94]

In 2008, the MOH proposed universal coverage for pediatric dentistry and the elderly population under the NHIL, [40] but this proposal did not pass the MOF and was not discussed by the government. A continued commitment to this issue was expressed later by the Director General (DG) of the MOH (in June 2009), who publicly expressed his views regarding the justification for including dental treatment in the NHIL (in addition to two other reforms he wanted to promote), mentioning the previous failed attempt to implement it. [95]

During 2008, a task force for narrowing socio-economic inequality established by the President of Israel in conjunction with the Minister of Welfare and Social Services delivered its recommendations, one of which was the inclusion of pediatric dentistry in the NHIL. [96] During that period, other entities and studies emphasized current inefficiencies and recommended including pediatric dentistry in the NHIL as well. [97-99]

*Incremental changes towards creating a window of opportunity*

In 2008, based on the information and arguments presented in these publications [38, 39], three civic organizations presented the HCJ with two petitions related to schoolchildren’s dental-health services. [41] They asked the court to issue an *order nisi* (temporary injunction) against the MOH’s provisions related to dental services for schoolchildren that treated these services as optional and delivered them in a discriminatory manner, meaning only through municipal jurisdictions that chose to provide them. Ostensibly, a positive decision of the court might have had serious implications, such as class action suits. Apparently, the state had an interest in avoiding a court ruling. Soon after the discussions in court began, the government announced that it was allocating three times the previous amount to budget for these services, but the discussions in court continued on matters beyond the budget. While the case was still being heard, some of the petitioners organized a coalition of 12 civic organizations to promote public dental services under the NHIL^b^. It included advocacy organizations and dental health provider organizations (such as the Dental Hygienists’ Association and the Association of Arab Dentists in Israel). [100] The Israel Dental Association did not join this initiative. The newly organized advocacy coalition sought to raise public awareness about this issue. Consequently, after many years with no bills in the Knesset promoting universal public coverage for pediatric dentistry, between 2008 and 2009, 15 private-member bills about dental care were presented in the Knesset [42], some of them specifically proposing including pediatric dentistry in the NHIL. [101] The policy network was very active and succeeded in raising the awareness of the public and politicians about the oral health issue and even launched maneuvers (such as the above mentioned petitions to the HCJ) that could change governmental incentives for providing and funding pediatric dentistry.

In March 2009, a senior official joined the MOH administration as a deputy director general (hereinafter: DDG), in a position with potential influence over the ministry policy. He moved from the position of senior researcher in a think tank devoted to applied policy research and published the above-mentioned papers that called for government intervention and served as the basis for the petitions to the HCJ. [38, 39]

***Entrance of a policy entrepreneur***

On April 7, 2009, after general elections in Israel and the replacement of the government, Rabbi Yaakov Litzman was named as a new Deputy Minister of Health (hereinafter: the DM or the entrepreneur). Litzman joined the coalition that established the new government, as a delegate from Yahadut Hatorah, a sectorial party that represents part of Israel’s ultra-Orthodox minority. According to his party’s coalition agreement with the ruling Likud party signed on April 1^st^ [43], the Prime Minister (of Israel’s 32^nd^ government) would hold the health portfolio throughout the Government’s term and a representative of Yahadut Hatorah would serve as sole deputy minister in the MOH^c^. The Prime Minister, the sides also stipulated, would empower the DM to act in his name in the Knesset and in the MOH in all ministry-related affairs within his competence and remit.

On March 6, 2009, less than one month prior to signing that coalition agreement, a two-page investigative article was published in *HaModia*, the main newspaper of the ultra-Orthodox Jewish population in Israel, [102] describing the severe situation in pediatric dentistry and citing the above-mentioned publication [38], and expressing the expectation that the MOH in general and the DDG in particular, would take substantial steps to rectify the situation. Nonetheless, even though Yahadut Hatorah representatives anchored demands regarding specific issues such as education and religious affairs in their signed agreement with the Likud party as a condition for joining the coalition and the government, [43] no demands were included regarding the healthcare system in general or pediatric dental care in particular. Moreover, a week after the new DM entered the MOH, he was interviewed and mentioned his plans for major policy changes in the ministry regarding various important issues, but not dental health services. [44] These events seem to indicate that the DM entered the Ministry with no agenda regarding the issue of pediatric dentistry.

In June 2009, the DM and the Director General of the MOH received a position paper from the DDG stressing the need to include pediatric dental care in the NHIL in a way that would ensure universal delivery of pediatric dental services. The submission of the paper stemmed from what might be interpreted as a window of opportunity for change. First, there was increased public awareness of and desire for such a change. Second, it was believed that the decision-makers in the MOF felt threatened by the ongoing litigation regarding dental services for schoolchildren and would be more responsive to new initiatives. Third, the HMOs had started to include zero co-pay services for children in selected age groups as part of their supplemental insurance and had already established a supply network with an appropriate geographic distribution and reasonable accessibility. Therefore, they could provide any dental services included in the basic basket of the NHIL nationally without delay. The fourth factor related to budget resources: 2010 was the third consecutive year in which an annual allocation of an increased budget designated for the addition of new technologies to the NHI basket of health services and medications was made. Some believed that the 2010 annual budget for new technologies might not be used in full for essential and life-saving technologies (LST) or services, so some of it could be earmarked for the inclusion of pediatric dental services under the Second Addendum of the NHIL. The basic assumption was that the DM had a mandate to promote his policy and could prioritize some portion of the MOH budget, and that such a decision would clear judicial review.

***Leveraging the window of opportunity***

The DM identified a window of opportunity, and on September, 2009 he expressed a [45] wish to include pediatric dentistry in the basket of services under the NHIL using part of the LST budget. From that moment on, he took firm action to promote a reform in which pediatric dental treatment would be included in the NHIL as a formal governmental policy, in accordance with the recommendations that appeared in various reports, publications [30, 38, 34-36, 94, 96-98] and parliamentary bills. [101] The DM received the full backing of his ministry’s senior officials as well as members of the policy network. Thus, he enjoyed the requisite support of professional experts and experienced civil-servants^a^ who were committed to the cause and willing to take responsibility for the process and its outcomes. The DM also held talks with the managements of the HMOs and IDA to explain his program and persuade them to agree to it.

Furthermore, given public awareness of the problem and the status of the DM in the government, he received backing for his initiative from his government peers: the Prime Minister, who held the health portfolio at the time, [46] the Bank of Israel, which published an analysis of the pediatric dental-care field supporting the MOH’s measures, [103] and ultimately even the President of Israel together with the Minister of Welfare and Social Services, who expressed, their support for the DM’s initiative, during a meeting with several representatives of a think tank. [104] The continuous efforts in coalition building for this reform and the amassing of political support paved the way for a positive outcome.

*Choosing a service-delivery model*

One of the important decisions regarding the design of the policy concerned the model by which pediatric dental services would be delivered under the NHIL. Presumably, this service, like all services covered under the Second Addendum of the NHIL, would be provided by the entities that the NHIL designated as the deliverers of basic services--the four HMOs. Prior to the enactment of the NHIL, the IDA objected to including the suggested dental package of services in the NHIL. [105] In 2009, the IDA supported public funding for pediatric dentistry and its inclusion in the NHIL, but objected to the proposed service-delivery model that initially was based exclusively on the HMOs. The IDA favored the establishment an independent authority that would deliver the dental health services under the NHIL exclusively (as a substitute for the HMOs) or the creation of an additional HMO or a special Corporation for Delivering Dental Services under the NHIL (CDDS), in addition to the existing four HMOs. [50]

From the IDA’s perspective, creating a special-purpose HMO or independent authority for dental care would allow the resulting entity to contract out most of its activity to the private clinics of the IDA’s members. The IDA also claimed that if the HMOs had full control of the service and no new HMO or company was established, citizens would forfeit the right to choose their dentist, materials and treatments would be locked in at a cost-cutting standard, access would suffer, and huge sums would flow into the HMOs’ coffers instead of directly to dental care. [52, 106]

The HMOs opposed this model, arguing they were already prepared to provide the service nationally at as high quality and accessibility as any other service in the NHIL. Politicians, legislators and members of the Coalition for Public Dentistry rejected the intention to allow private, for-profit corporations, such as the insurance industry, to be providers of public healthcare services under the NHIL. [52, 107]

During the last stages before adoption, the reform plan was presented to the Health Council, a statutory advisory panel for the Minister of Health that includes the main stakeholders in the entire healthcare system as well as public representatives. The council endorsed the proposed reform by a majority vote, emphasizing the need to assign service delivery to the HMOs. Most of the participants rejected the model that would establish a special CDDS. [108]

However, as an interest group, the IDA succeeded somewhat in exploiting its strength vis-à-vis the politicians, particularly in advancing its service model. Remarks by the DM to the chair of the IDA during a debate about the reform in the Knesset Labor, Welfare, and Health Committee support the claim that the IDA attempted to influence politicians as well as government ministers. [107] The MOF and IDA enjoyed a commonality of interests regarding the service-delivery model. The MOF had long sought to augment the healthcare system with a fifth HMO, [51], believing that this would enhance competition among the HMOs. Thus, some have argued that the proposed reform was an opportunity for the MOF to create a precedent, in the hopes that later on it could expand the CDDS’s services into additional fields of medicine and compete with the existing four HMOs [52].

As for the MOH, most of its relevant senior officials were concerned about the entry of another as-yet-unproven actor into this area, and they strongly appreciated the ability of the HMOs to provide the dental services. However, the DM deferred to the CDDS model and acceded to the inclusion of a special-purpose CDDS in the NHIL. [107] His reasoning might have included both his willingness to minimize the possibility of damage to private dentists and a desire to increase the free choice of dental providers. It may also have involved a pragmatic calculation, meaning, a belief that, given the popularity of this model among government members, including the Prime Minister, the reform would not receive the government’s approval unless the creation of a CDDS were permitted.

The wording of a government resolution of May 4, 2010 connected the timeframe of the inclusion of pediatric dental care for children up to age eight in the Second Addendum of the NHIL to the operation of the new entity (CDDS) that would provide services mostly by private, self-employed dentists. [109] The DM objected to that wording. He argued that the complex process involved in approving and establishing a special-purpose company might take a great deal of time, delaying the reform, and there was no reason to prevent Israel’s children from immediate access to publicly funded dental services through their HMOs. Ultimately, the DM and his supporters succeeded in uncoupling the implementation of the reform from the establishment of the special CDDS, so that children could begin receiving dental services immediately through their HMOs starting July 1, 2010, regardless of when the MOH approved any additional corporation.

Accordingly, on June 16, 2010 the government submitted a bill to the Knesset [110] which included, among other subjects, a model for providing pediatric dentistry under the NHIL via the four existing HMOs, as well as CDDS operating under Israel’s Companies Act 1999 [111] (which refers to for-profit as well as non-profit corporations), conditional on the MOH’s approval of them (the bill did not limit the number of such corporations). However, as will be described later, the legislators made a last minute change in the proposed model.

***Finding a budget source for the reform***

The estimated cost of the first phase of the reform was 115 million NIS. The MOF demanded that 65 million NIS of that budget should come from the MOH’s resources (the rest should be based on the national budget). The DM announced that 65 million NIS for that purpose would be transferred from the 2010 annual budget (415 million NIS) for new medicines and technologies. This decision was backed later by government resolutions. [112]

The Israel Medical Association (IMA), the healthcare-policy elite in academia, several patients’ organizations, and others vehemently opposed the decision to obtain some of the requisite annual budget from an allocation intended for the inclusion of new life-saving technologies in the NHI. The resistance received massive media coverage. Its background was twofold: the desire to leave the amount of new technology that could be added to the NHI that year untouched, and the fear of a precedent that would allow the MOH to divert the new technologies budget to other uses in the future. Thus, the criticism involved not the inclusion of pediatric dental care in the NHI per se, but the way the MOH chose to fund it. [47, 113] Nevertheless, since other funding sources were not available, this objection had the potential to derail the suggested reform. One of the main opponents of this initiative, the IMA, submitted a petition to the HCJ against the government resolution to use part of the LST budget as a funding source for the reform. [47]

Civic organizations were divided in their attitudes towards that issue. Some of them initiated (independently) two petitions to the HCJ against the MOH decision that could negatively affect a designated budget. [47] Others supported the MOH position and joined the court as *amicus curiae* (February 2010). [48] They stressed the importance of the DM’s program, calling it an historic move that would correct the injustice of excluding pediatric oral and dental health when the NHIL was enacted. To promote consensus about this matter, the MOH and the board of the Israel National Institute for Health Policy and Health Services Research convened the Institute’s Policy Forum, consisting of several dozen senior healthcare executives and health policy analysts to talk it over. [114] Nevertheless, as described later, discussions in the HCJ on that issue continued.

***The role of the judicial authority***

Two events demonstrate the role of the judiciary in this process. The first event describes a situation in which what seems to be a substantial involvement of the Court, through a nullification of a government resolution by the judicial authority, had actually a very limited impact on the implementation of the policy. The second demonstrates minimal involvement of the judicial authority, but one that could have a substantial impact on the government’s motivation to change an existing policy.

*The impact of a petition to the HCJ on policy design: The case of a budget source for the reform*

As mentioned, given the opposition to the suggested funding source for the reform, several NGOs and the IMA appealed, independently, to the HCJ [47] to forbid the state from using the new technologies budget to fund the reform. In February 2010, the HCJ responded by issuing an *order nisi* instructing the state to explain why it should not rescind its decision to subtract 65 million NIS from the technologies budget. The HCJ’s final decision, [49] handed down in May 2010, voided the December 2009 government resolution that earmarked this money to supplement the NHI services for pediatric dental care. Nevertheless, the HCJ did not address the appropriateness to allocate the government’s designated LTS budget to fund pediatric dental care. Instead, it focused its criticism on the procedure under which the proposed pediatric dental services for children were supposed to be included in the basic basket of services supplied under the NHIL. The court clarified that in this case the government could not use the ordinary procedure for expanding new services to be included in medical areas listed in Section 6 of the NHIL. In that case, after obtaining the approval of the Minister of Finance and the government, an order signed by the Minister of Health is sufficient. However, since the area of ‘dental health’ is not one of the medical areas mentioned in Section 6, the government decision could be implemented only through one of two legal procedures: 1) First, expanding the list in Section 6, through an order signed by the Minister of Health, after receiving the approval of the Minister of Finance and the Knesset’s Committee for Employment, Welfare and Health, and then add pediatric dental services to the 2^nd^ Addendum of NHIL through an order signed by the Minister of Health, based on approval of the Minister of Finance and the government; or 2) Through a legislative change in NHIL that will add a new area of “dental health” to Section 6 of the NHIL.

Ultimately, the government implemented the HCJ order fully. First, as an immediate step, with the approval of the Minister of Finance and the Knesset Labor, Welfare, and Health committee, in his capacity as the Minister of Health, the Prime Minister issued an order to add a new area of dental health to Section 6 of the NHIL. [115, 107] Since the committee decided on a time limitation for its approval, the order was made as a temporary provision valid until November 30, 2010. At the same time, after receiving approvals from the Minister of Finance and the government, [116] another ministerial order [53] was issued to add the list of services included in the reform to the Second Addendum of the NHIL beginning July 1, 2010. Several weeks later, these steps were followed by a permanent legislative change that included all aspects of the reform including, among others, the change in Section 6 of the NHIL [29] that added the area of dental health and included dental health for children up to 8 years old in the Second Addendum of the NHIL. These measures eliminated the only judicial technicality that had impeded the reform, allowing the DM and his associates in the MOH to make progress toward its implementation including the use of the 65 million NIS from the LST budget as a funding source, as originally planned.

A financial agreement between the MOH and MOF was signed to ensure the successful implementation of the reform in three stages in addition to the immediate one, gradually increasing the eligibility of children up to 14 years old for the services by July 2013 (including the necessary funding from the state budget). The government approved the agreement. [54]

As described above, in this case, the petitions to the HCJ nullified a government resolution, but actually it had minimal impact on the policy adopted. The petitioners did not succeed in preventing the government from using a portion of the LST budget as a funding source for the first stage of the pediatric dentistry reform, as initially planned.

*The impact of a petition to the HCJ on motivations for a policy change: The case of dental services to schoolchildren*

As mentioned earlier, in 2008 several civic organizations submitted a petition to the HCJ claiming the MOH was violating the NHIL regarding preventive dental services to schoolchildren (as listed in the Third Addendum). On May 17, 2009, after the state made a large increase in the budget for this service [117] and while the discussions in court were still continuing, the government changed and the above-mentioned policy entrepreneur joined the MOH. The ministry was already discussing the possibility of including dental services under the NHIL and adopted it as important part of its agenda. Given this situation, the state advised the court that it also intended to implement a reform in pediatric dental-care treatments (restorative and preventive treatments) and include them in the basic services under the Second Addendum to the NHIL, meaning, they would be provided universally to children by the HMOs as part of the Second Addendum of the NHIL. They also stated that concurrently the MOH’s responsibility for preventive dental health of all schoolchildren under the Third Addendum of the NHIL would be implemented widely, through the local authorities. As the litigation continued, in September 17, 2009 the justices stated that the HCJ would monitor the implementation of the state’s announcement and ordered the state to present an updated statement in January 2010. The reform had not been implemented by January 2010. The court gave the government an extension. Only on September 15, 2010, approximately three months after the implementation of the reform, did the HCJ announce its final decision. It explained that given the fact that the state had budgeted 30 million NIS for preventive dental services for schoolchildren, was working to provide them through all of the local authorities, had included dental services for children as an integral part of the Second Addendum of the NHIL and was committed to expanding the program to all children up to 14 years old, "Under these circumstances, the circumstances that existed at the time of the filing of the petitions have changed, and the appeals have been exhausted.... therefore, they are annulled, with no order for costs." [55]

The HCJ’s statements and the fact that the court tracked the evolution of the reform and denied the petition only after it was clear that the reform had been implemented to all children might indicate that the court attitude was perceived as a looming threat for government officials, especially in the MOF. It is reasonable to assume that, in addition to other reasons, to avoid a far-reaching court ruling on the matter, they favored the reform offered by the MOH.

***The impact of the legislative authority***

*Last-minute change in the service-delivery model*

The struggle over the service-delivery model did not end with the government resolution. Given that the NHIL approved only the four HMOs as the providers of health services included in the Second Addendum, the existence of a new entity, such as the CDDS, had to be included as an amendment to the NHIL. In the course of the debate over that amendment, the Knesset Labor, Welfare, and Health Committee decided to amend the government’s legislative memorandum so that the CDDS would be approved, but only after changing their designation from *“a corporation operating under the 1999 Israel’s Companies Law”* (which includes for-profit as well as non-profit corporations) to *“public-benefit corporations”* (limited to non-profit corporations). This step was the result of strong pressure from committee members and various civic organizations that objected passionately to the possibility of allowing for-profit commercial entities to be public insurers responsible for providing basic NHI services.

On July 22, 2010, the government secretary announced [118] that the Knesset had approved an amendment allowing the recognition of not-for-profit entities that would provide pediatric dental-health services. He added that the Knesset adopted the Prime Minister’s proposal to stimulate competition in dental care, reduce prices and improve service in this area. He also mentioned that the right to choose a provider is a fundamental right.

Since July 1, 2010, the four HMOs (the main service providers of the NHIL) have provided pediatric dental services. So far, very few non-profit organizations have applied for recognition as CDDS, and none has been approved by the MOH. For-profit corporations such as insurance companies that theoretically could have been candidates as CDDS are not eligible to apply.

As described, the policy entrepreneur succeeded in implementing the first phase of a very meaningful reform. As mentioned, the next phases should have been implemented gradually over the following three years.

***Limiting the entrepreneur's power and changing the incentives***

The sustainability of a reform, even when the ruling party considers it a remarkable public policy achievement, [119] cannot be taken for granted when circumstances change and key actors are replaced. Some members of the network may lose the incentive or ability to sustain their accomplishment. This combination of factors may upset the equilibrium among the forces that comprise the network and allow new actors in key positions to revise priorities and stop the reform in midstream, even if the positions of other actors and their relative strengths do not change. This is exactly what happened in our case study.

As of early 2013, the pediatric dental care reform was in effect for ages 0-12, and the final implementation stage (expanding eligibility for pediatric dental service under the NHI to children ages 12–14) was to go into effect in several months (July 2013). This course was altered when a new minister took over in the MOH, due to general elections in Israel and the establishment of a new government. This time Yahadut Hatorah was not a member of the coalition and had no representation in the government. In discussions between the MOH and MOF on the 2013 and 2014 budget, the incoming Minister of Health and MOF officials agreed to cancel the final stage of the reform and prevented the addition of the 274,000 youngsters in this age group [56] to those eligible for NHI-financed dental treatments. Doing so disrupted the full implementation of the three-year agreement that both ministries had signed in 2010 that had secured and financed the final phase of the reform approved by the previous government. [54] The turnaround reflected a change in the balance of forces within the policy network since the network’s endorsement of the reform. Apparently, the new Minister of Health gave little priority to the continuation of her predecessor’s policy on pediatric dental care. It also appears that she had little interest in oral health, given that, in contrast to opinions expressed by senior officials in the MOH and academia, she cancelled the regulations that permitted water fluoridation in Israel (given to more than 70% percent of the population), [120] without allocating substantial resources for other national-level prevention plans and ensuring their impact and successful implementation to avoid a deterioration in children’s oral health.

As for the former DM (the entrepreneur), on July 21, 2014, as a member of the Knesset with no governmental position, and backed by 40 other MKs, he proposed a private bill to include dental health services for children up to 18 years of age in the NHIL. [57] This initiative failed. It was rejected by the government [58] and not approved by the Knesset. This unsuccessful initiative was hardly mentioned in the media and did not result in any substantial public response.

In the years since the onset of the reform the relative power of some of the members of the network had changed. With the elimination of the petition to the HCJ, [55] the possible threat to the government was removed. The inclusion of a special CDDS among the providers of services in the NHIL had already been legislated, albeit in a not-for-profit form. Hence, the attempt to encourage meaningful competition between HMOs and CDDS had exhausted itself. Apparently, the MOF no longer had an incentive to complete the reform by funding the last stage of implementation, prior commitment or not.

On May 14, 2015, Israel changed governments again. Now, as two years before, Yahadut Hatorah joined the coalition and signed a coalition agreement with the ruling Likud party. [66] The agreement assured them a deputy minister in the MOH with a status resembling that established in the previous accord (later on, the position was upgraded to Minister of Health^d^). This time, however, in contrast to the previous one, the accord included demands and commitments for the implementation of reforms in two matters related to the dental healthcare system: the implementation of the last stage of pediatric dental-care reform (expanding the entitlement to children up to 14 years old) by January 1, 2016, and general intentions to extend it gradually even beyond the eligibility ages previously specified (15-18 years) by 2020. Indeed, since January 2016 the last stage of the reform that the previous government cancelled has been accomplished, as originally planned by the entrepreneur, after receiving the government’s approval [59] and completing the necessary legislative procedures. From that time on, children up to 14 years old have been eligible for pediatric dental-care under the NHIL, as was originally intended under the reform. In addition to the pediatric dental services provided by the HMOs (under the Second Addendum), preventive check-ups in oral health as well as health-education lessons in oral health have been expanded and are provided to schoolchildren by most of the local authorities in Israel under the Third Addendum of the NHIL. [60]

***********

**Table 1: Main Milestones in the emergence and implementation of the pediatric-dentistry reform**

|  | **Event** | **Date** | **Main actors** |
| --- | --- | --- | --- |
| 1 | A state investigative commission report recommends enactment of a NHIL in Israel and inclusion of dental health services for children up to 18 years by law (among other services) | August 20, 1990 | Government |
| 2 | Initial government intention to include dental health services (including restorative and prosthetic dentistry) for children and the elderly population in a NHIL (**government #24**) | February 17, 1992  NHIL Bill 1992 | Government + legislators |
| 3 | A new government bill is submitted to the Knesset (including preventive dental-services for schoolchildren) (**government #25**) | June 30, 1993  NHIL Bill 1993 | Government + legislators |
| 4 | NHIL enactment. Restorative and prosthetic dentistry for children and the elderly were not included (preventive dental-services for schoolchildren are included without clear definition of the services) | June 15, 1994 | Government + legislators |
| 5 | Early academic publications and policy papers regarding national policy on dental-health services | 1996 -1999 | Researchers in academic institutes and think tanks |
| **Government #31 Begins its Term** | | May 4, 2006 |  |
| 6 | Two policy analysis publications bolster network activities | July 2007  December 2007 | Researchers in academic institutes and think tanks |
| 7 | MOH prepares a plan to include dentistry in the NHIL. It did not pass the MOF and was not discussed by the government | February-March, 2008 | MOH civil-servants^a^ |
| 8 | Submission of two petitions to the HCJ (1^st^ petition series), based on arguments appeared in the above-mentioned publications regarding universal coverage of pediatric dentistry | March 12, 2008  March 6, 2008 | Civil organizations and judiciary authority |
| 9 | Establishment of the Coalition for Public Health Dentistry, by 12 civil organizations | November 27, 2008 | Civil organizations |
| 10 | Submission of private bills by legislators to include pediatric dentistry in the NHIL | June 23, 2008-April 1, 2009 | Legislators |
| **Government’s Replacement** (**Government #32**) | | March 31, 2009 |  |
| 11 | A new Deputy Minister enters the MOH (apparently, with no prior agenda regarding dentistry) | April 6, 2009 | Politicians |
| 12 | The DDG delivers a policy proposal to the DM and Director General regarding a policy change in pediatric dentistry | June 3^rd^, 2009-July 5^th^, 2009 | MOH civil-servants |
| 13 | DG of MOH declares intention to add dental services to NHIL | June 3, 2009 | MOH civil-servants |
| 14 | First announcement of the DM’s intention to include dental health in the NHIL (2^nd^ addendum), using a budget designated for another purpose (LST) | September 9, 2009 | Entrepreneur |
| 15 | The state informs the HCJ about its intention to include pediatric dentistry as a universal right in the 2^nd^ addendum of NHIL | September 17, 2009 | Politicians and judiciary authority |
| 16 | MOF+IDA attempt to include a CDDS as provider of pediatric dental services under the NHIL | September 2009-July 2010 | Government + civil-servants + professional interest group |
| 17 | Government resolution (#1064) to take 65 million NIS from the budget designated for high-technology life-saving medications to fund the reform | December 14, 2009 | Entrepreneur + government |
| 18 | Submission of three petitions to the HCJ (2^nd^ petition series) regarding the funding source of the reform | December 15, 2009  December 17, 2009  December 29, 2009 | Professional interest group (IMA); civil society organizations; judiciary authority |
| 19 | Civil organizations join - as *amicus curiae -* in the petition to the HCJ on the reform budget | February 1, 2010 | Civil society organizations and judiciary authority |
| 20 | Court resolution on the funding source of the reform (2^nd^ petition series): the government resolution enabling cutting the designated budget in order to fund the pediatric dental reform is nullified due to procedural problems | May 20, 2010 | Judiciary authority |
| 21 | Two ministerial orders are issued (in accordance with the procedure ordered by the HCJ) enabling reform implementation | June 14, 2010 | Government |
| 22 | 1^st^ stage of reform is implemented entitles children up to 8 years old to receive dental services through the HMOs as part of the basic basket of health services under the NHIL. | July 1, 2010 | Legislators + Government +HMOs |
| 23 | An amendment adds the area of dental health to the services covered by the NHIL. The amendment allows the establishment of a non-profit CDDS as a provider of these services | July 26, 2010 | Legislators + government |
| 24 | Resolution of the HCJ on the 1^st^ petition series (regarding dental services to schoolchildren) –due to activities made by government regarding pediatric dentistry - petitions annulled | September 15, 2010 | Judiciary authority |
| 25 | Expanding the entitlement for pediatric services under the NHIL to children up to 10 (2^nd^ stage of the reform implementation) | July 1, 2011 | Government |
| 26 | Expanding the entitlement for pediatric services under the NHIL to children up to 12 (3^rd^ stage) | July 1, 2012 | Civil-servants at MOF+MOF |
| **Government’s Replacement (government #33)** | | March 13, 2013 |  |
| 27 | The last stage of reform is not implemented due to a decision made by new Minster of Health and MOF officials | July 1, 2013 | Government + civil-servants at MOF |
| 28 | Private bill to add dental health services to children up to 18 years is submitted to the Knesset by the former DM and other 40 MKs – it failed | July 21, 2014 | Entrepreneur + legislators |
| 29 | Coalition accord for the formation of the 34^th^ government between Likud and Yahadut Hatorah guaranteeing the latter a DM of Health and a commitment to implement the last stage of the pediatric reform | May 3, 2015 | Politicians |
| **Government’s Replacement** (34^th^ government) | | May 14, 2015 | Politicians |
| 30 | Last stage of the reform is implemented (2.5 years later than the original plan). Children up to 14 years are entitled to received dental treatments through their HMOs as an integral part of the NHIL | January 1, 2016 | Entrepreneur + government |

******
